# Expression of NELL2/NICOL-ROS1 lumicrine signaling-related molecules in the human male reproductive tract

**DOI:** 10.1186/s12958-023-01175-6

**Published:** 2024-01-02

**Authors:** Daiji Kiyozumi

**Affiliations:** 1 Japan Science and Technology Agency, 7, Gobancho, Chiyoda-ku, Tokyo, 102-0076 Japan; 2 Research Institute of Environmental Medicine, Nagoya University, Furo-cho, Chikusa-ku, Nagoya, 464-8601 Japan

**Keywords:** Lumicrine, Caput epididymis, Initial segment, NELL2, NICOL, ROS1, Sperm maturation, Human

## Abstract

The maturation of spermatozoa is a regulated process, influenced by genes expressing essential secreted proteins in the proximal epididymis. Recent genetic studies in rodents have identified the non-sex steroidal molecular signals that regulate gene expression in the proximal epididymis. Germ cells in the testis secrete ligand proteins into the seminiferous tubule lumen The ligand proteins travel through the male reproductive tract lumen to the epididymis, where they bind to receptors, triggering the differentiation of the luminal epithelium for sperm maturation. It is, however, not fully unveiled if such a testis-epididymis trans-luminal signaling mechanism exists in other species, especially humans. In the present study, the rodent-type testis-epididymis trans-luminal signaling in the human male reproductive tract was evaluated in a step-by-step manner by analyzing testis and epididymis gene expression and signaling mediator protein function. There was a significant correlation between the epididymal expressions of mouse genes upregulated by the trans-luminal signaling and those of their human orthologs, as evaluated by the correlation coefficient of 0.604. The transcript expression of *NELL2* and *NICOL* encoding putative ligand proteins was also observed in human testicular cells. In vitro experiments demonstrated that purified recombinant human NELL2 and NICOL formed a molecular complex with similar properties to rodent proteins, which was evaluated by a dissociation equilibrium constant of 110 nM. Recombinant human NELL2 also specifically bound to its putative receptor human ROS1 in vitro. Collectively, these findings suggest that the rodent-type testis-epididymis secreted signaling mechanism is also possible in the human male reproductive tract.

**Supplementary Information:**

The online version contains supplementary material available at 10.1186/s12958-023-01175-6.

## Introduction

Production of competent gametes is the prerequisite for successful reproduction. In aquatic vertebrates, spermatozoa generated in the testes are fully capable of fertilization. In terrestrial vertebrates such as mammals, spermatozoa generated in the testicular seminiferous tubules are still immature and need further functional maturation to acquire full fertilizing ability. The maturation of spermatozoa occurs at the post-testicular level; the testicular spermatozoa are transported via the efferent duct to the epididymis, where they become functionally mature. The epididymis is a highly coiled epithelial duct constituting a part of the sperm transport route. The spermatozoa develop the fertilizing ability as they pass through the lumen of the epididymis. In the epididymis, various proteins including secreted protein families such as β-defensins, lipocalins, and cystatin-related epididymal spermatogenic (CRES) proteins are specifically expressed to modify the fertilizing ability of spermatozoa [1–3].

The mechanisms of sperm maturation in the epididymis are orchestrated by the testes. Endocrine control of epididymal gene expression and function is facilitated by sex steroids such as androgens and estrogens synthesized by Leydig cells within the testes. These sex steroids are transported through the bloodstream to the epididymis, where they interact with their receptors, thereby modulating gene expressions and cellular functions, as shown by gene targeting strategies [4, 5]. In addition to the endocrine action of sex steroids, a mechanism involving the transmission of signals by non-steroidal factors through the reproductive tract has been identified in rodents. When solely ligating the efferent duct while leaving the testes intact, the differentiation of the luminal epithelial cells of the initial segment (IS) epididymis and subsequent induction of gene expression becomes disrupted [6, 7]. This experimental evidence strongly indicates the presence of a signaling mechanism, through which the testes regulate cellular processes in the epididymis via the efferent duct, at least in rodents. Since this type of secretion signaling between the testes and epididymis acts through the lumen, it has been referred to as “lumicrine,” a terminology introduced by Barry T. Hinton [8].

**Fig. 1:**
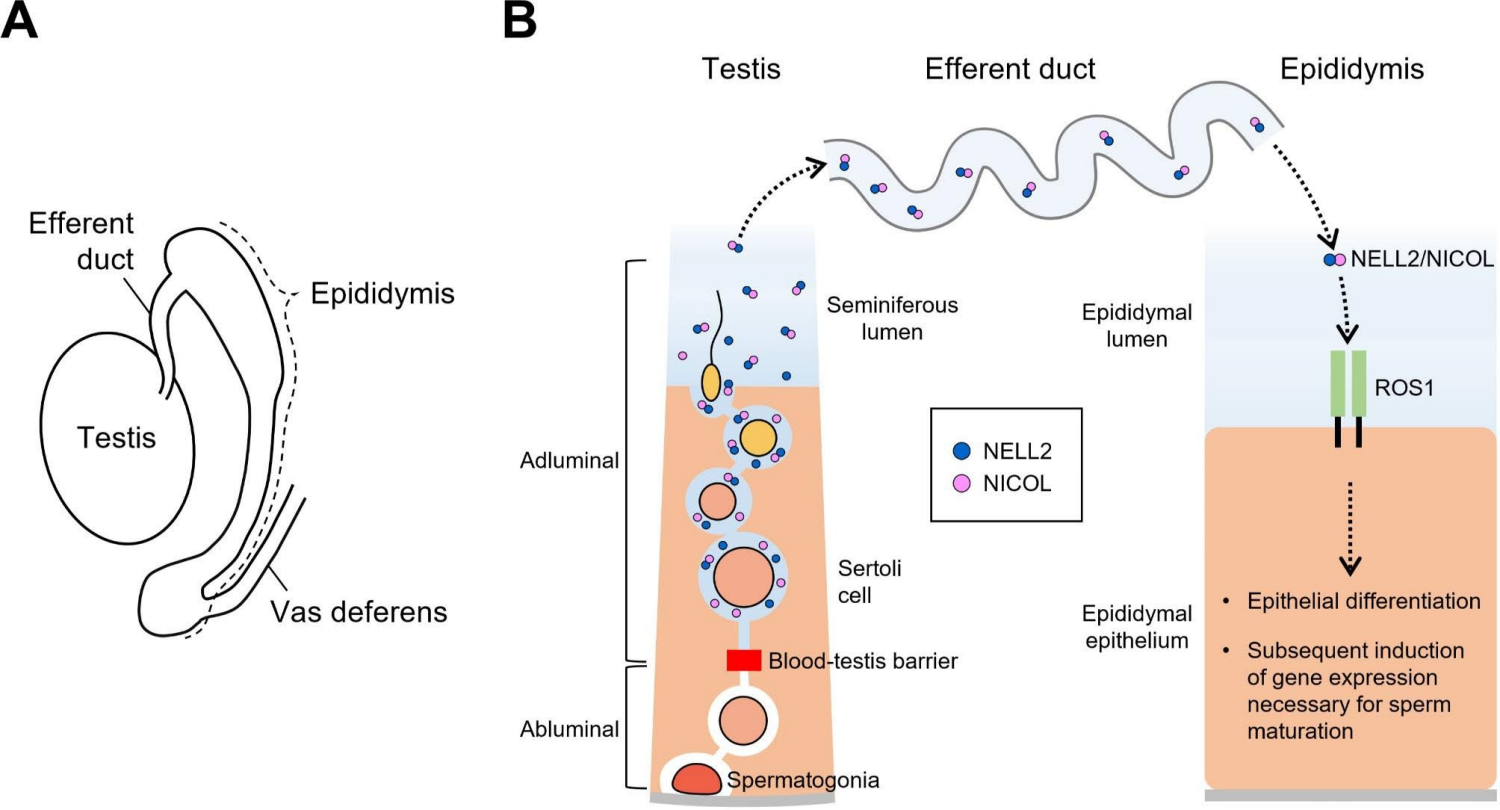
A schematic representation of testis-epididymis trans-luminal signaling in rodents. (**A**) A schematic representation of rodent male reproductive tract. (**B**) A schematic representation of testis-epididymis trans-luminal signaling lumicrine. In the testicular seminiferous tubule, lumicrine factors such as NELL2 and NICOL are secreted from germ cells located inside the blood-testis barrier. The secreted ligand proteins go trans-luminally through the efferent duct to the epididymis. In the epididymis, the testis-derived ligands bind to the receptor ROS1 on the apical surface of the epididymal luminal epithelium. Upon ROS1 activation, genes necessary for epididymal differentiation and sperm maturation are induced

Utilizing reverse genetic analyses in genetically modified mice, key components involved in this testis-epididymis signaling network have been identified. Neural EGF-like like 2 (NELL2) and the NELL2-interacting cofactor for lumicrine signaling (NICOL) serve as ligand proteins secreted by the germ cells within the testes into the reproductive tract lumen [9, 10]. These ligand proteins traverse the reproductive tract lumen to reach the epididymis, where they bind to the receptor-type tyrosine kinase ROS1 expressed in the IS [11–13]. Upon ligand binding, ROS1 activates downstream intracellular signaling pathways [14, 15]. These pathways lead to the induction of luminal epithelial cell differentiation in the IS region and the expression of various genes including β-defensins, lipocalins, CRES/cystatins, proteases, and other secreted proteins essential for sperm maturation [1–3, 9]. The testis-epididymal trans-luminal signaling in the male reproductive tract is thus apparent in rodents as schematically summarized in Fig. 1.

The tall pseudostratified luminal epithelium of the IS triggered to differentiate by the trans-luminal secreted signaling is a prominent histological feature characteristic of rodents [6, 7, 9, 10, 14]. The testis-epididymis trans-luminal signaling system and its regulation of epididymal functions in humans have not been fully investigated, because the human epididymis lacks anatomical features such as the prominent epididymal IS in rodents [16, 17], implying another mechanism distinct from murine lumicrine signaling. However, the tall luminal epithelium of the rodent epididymal IS has not been shown to be the universal indicator of lumicrine-regulated epididymal function including sperm maturation across species although it is a good indicator of lumicrine signaling in rodents. Therefore, it is necessary to evaluate carefully the possibility of testis-epididymis trans-luminal signaling in humans based also on non-histological data.

The bioinformatic approaches using next-generation sequencing data have significantly enhanced the capabilities of gene expression analyzes and supplement histological approaches. By conducting genome-wide analyses, in addition to individual gene expression, the regulatory mechanisms working behind the specific biological processes can be investigated. In addition to such gene expression analysis, protein function analyzes are also valuable in characterizing biological processes. In the present study, the potential testis-epididymis communication through the male reproductive tract in humans was evaluated by transcriptome and protein biochemical analyzes.

## Materials and methods

### Epididymal anatomy

In the mouse transcriptome analyses, the IS and caput were combined, and such a tissue dissection was indicated by the description “IS-caput” throughout the manuscript.

### Transcriptome analyses

The following human and transcriptome datasets publicly available were downloaded from the NCBI Gene Expression Omnibus website (https://www.ncbi.nlm.nih.gov/geo/): GSE150852, RNA-seq data of human epididymis [18]; GSE133920, RNA-seq data of WT and *Nell2* knockout (KO) mouse caput epididymis [9]. To convert gene accession numbers into gene names, the DAVID Bioinformatics Resources website (https://david.ncifcrf.gov/home.jsp) was used. The transcriptome data were incorporated into Microsoft Excel software for further analysis. The human testis single-cell RNA-seq dataset, which contains information about gene expression in individual cells and cell population clustering, was downloaded from the Mendeley Data website (https://data.mendeley.com/datasets/kxd5f8vpt4/1) [19]. The dataset was imported into Loupe Browser software 6.1.0 (10x Genomics, Inc.) to extract the expression profiles for human *NELL2* and *NICOL* transcripts. Human orthologs of mouse genes were identified on the NCBI Gene website (https://www.ncbi.nlm.nih.gov/gene/).

### Plasmid construction

cDNAs encoding C-terminally 6×His-tagged, human NELL2, C-terminally 6×His-tagged human NICOL, and C-terminally 8xHis and Rho1D4-tagged human ROS1 ectodomain were cloned into a pCAG vector containing the CAG promoter and a rabbit globin poly(A) signal. A C-terminally 6×His-tagged cartilage oligomeric matrix protein (COMP) expression plasmid was constructed previously [9].

### Protein purification

Human 293-F cells (Invitrogen, #R79007), a derivative of HEK293T cells, was used for recombinant protein expression. 293-F cells were cultured in suspension in HE400/AZ chemically defined medium (Gmep Inc., Japan, #HE400AZ-0010). A C-terminally 6×His-tagged COMP was expressed in 293-F cells and purified from the conditioned medium as described previously [9]. A C-terminally 6×His-tagged recombinant human NELL2 protein and C-terminally 6×His-tagged human NICOL protein were transiently expressed in 293-F cells by transfecting expression plasmids using a Gxpress 293 Transfection Kit (Gmep Inc., #GX293-RK-0010) according to manufacturer’s instructions. After 3 to 5 days of culture, the conditioned medium was harvested by brief centrifugation and the proteins were precipitated by ammonium sulfate at a final concentration of 80%. The precipitate isolated by filtration was then dissolved in 20 mM Tris-HCl pH 8.0, 30 mM imidazole, and 1 mM phenylmethylsulfonyl fluoride and loaded onto nickel affinity resin (Qiagen, #30,210). After washing with 20 mM Tris-HCl pH 8.0, 30 mM imidazole, and 150 mM NaCl, the bound protein was eluted with 300 mM imidazole pH 8.0, desalted using a PD-10 gel filtration column (Cytiva, #17,085,101) pre-equilibrated with phosphate-buffered saline (PBS). Recombinant 6×His-tagged human NELL2 and 6×His-tagged human NICOL were validated by SDS-PAGE under reducing conditions and subsequent immunoblot detection with anti-NELL2 antibody and anti-NICOL antibody, respectively. The concentrations of purified recombinant proteins were determined with the Pierce™ BCA Protein Assay Kit (Thermo Fisher Scientific Inc., #23,225) relative to bovine serum albumin (BSA) as the standard. The absorbance at 560 nm was monitored using an iMark microplate reader (Bio-Rad Laboratories, Inc.). Purified proteins were stored at − 80 °C until use.

### Immunoblot analyses

Proteins were separated by SDS-PAGE under reducing conditions, and blot onto polyvinylidene difluoride membrane using Trans-blot turbo system (Bio-rad, #1704150J1). The membranes were incubated with a blocking solution (3% BSA and 0.05% Tween20 in Tris-buffered saline) for 30 min, then with primary antibodies diluted in the blocking solution overnight. The bound antibodies were detected by following incubation with peroxidase-conjugated secondary antibodies and chemiluminescent reaction using Chemi-Lumi One Super (Nacalai Tesque, #02230). The chemiluminescent signals were detected using Amersham ImageQuant 800 (Cytiva). Antibodies used were as follows: rabbit polyclonal anti-NELL2 antibody (Proteintech, #11268-1-P), rabbit polyclonal anti-NICOL antibody (Atlas antibodies, #HPA052447), mouse monoclonal anti-Rho1D4 antibody (Cube biotech, # 40,020), peroxidase-conjugated goat polyclonal anti-rabbit IgG (Jackson Immunoresearch, #111-036-045), and perpoxydase-conjugated goat polyclonal anti-mouse IgG (#115-036-062, Jackson Immunoresearch). Uncropped immunoblot images were provided as Supplementary Material 1.

### Protein interaction analyses

To investigate protein interactions with human ROS1, a total of 100 µg of each purified protein was conjugated with a 25-µL bed volume of NHS-activated agarose (Thermo Fisher Scientific Inc., #26,200). For NELL2 pulldown, purified recombinant NELL2 was applied to protein-conjugated beads, and for ROS1 pulldown, ROS1 ectodomain tagged with 8xHis and Rho1D4 was transiently expressed in 293 F cells, and the transfected cells were lysed with lysis buffer. Cell lysate (1 mL of 1 mg protein/mL) was mixed with a 25-µL bed volume of protein-conjugated agarose beads and the mixture was incubated overnight at 4 °C with gentle rotation, washed three times with 1 mL of lysis buffer, and subjected to SDS-PAGE to separate the bound proteins for subsequent immunoblot analysis. Anti-NELL2 antibody and anti-Rho1D4 antibody were used to detect NELL2 and ROS1 ectodomain, respectively.

To determine kinetic parameters, a surface plasmon resonance assay was carried out by immobilizing purified human NELL2 protein onto a series S sensor chip CM5 (Cytiva, #29,104,988) as a ligand. Purified human NICOL protein dissolved in PBS was loaded as an analyte and its association and dissociation kinetics were monitored using a Biacore T200 instrument (Cytiva). The association/dissociation dynamics of NICOL to NELL2 at increasing concentration were monitored to determine the association rate constant *k*_a_, dissociation rate constant *k*_d_, and dissociation equilibrium constant *K*_D_.

### Statistical analysis

All experiments other than those using publicly available transcriptome data were repeated biologically at least thrice, and similar results were obtained. Student’s *t*-test *p* values and correlation coefficient r values were calculated with Microsoft Excel 2019 (Microsoft Corporation).

### Ethical approval

Not applicable.

### Drawings

Schematic drawings were generated using Microsoft PowerPoint software.

### Data availability

Transcriptome data supporting the results of this study are available at the NCBI GEO under accession numbers GSE150852 and GSE133920. The single-cell RNA-seq data supporting the results of this study are available at the Mendeley Data website (URL: https://data.mendeley.com/datasets/kxd5f8vpt4/1).

## Results

### Bioinformatic characterization of human epididymis gene expression

In the murine epididymal IS, many genes are regulated under the control of testis-epididymis trans-luminal signaling; such genes are downregulated in the epididymal IS of *Nell2* KO, *Nicol* KO, *Ros1* KO, *W*/*Wv*, or efferent duct-ligated mice in which testis-epididymis trans-luminal signaling is genetically or experimentally ablated [9, 10, 20]. To characterize gene expression in the human epididymis based on findings in mice, human genes orthologous to mouse genes expressed in the IS-caput epididymis are analyzed.

**Fig. 2:**
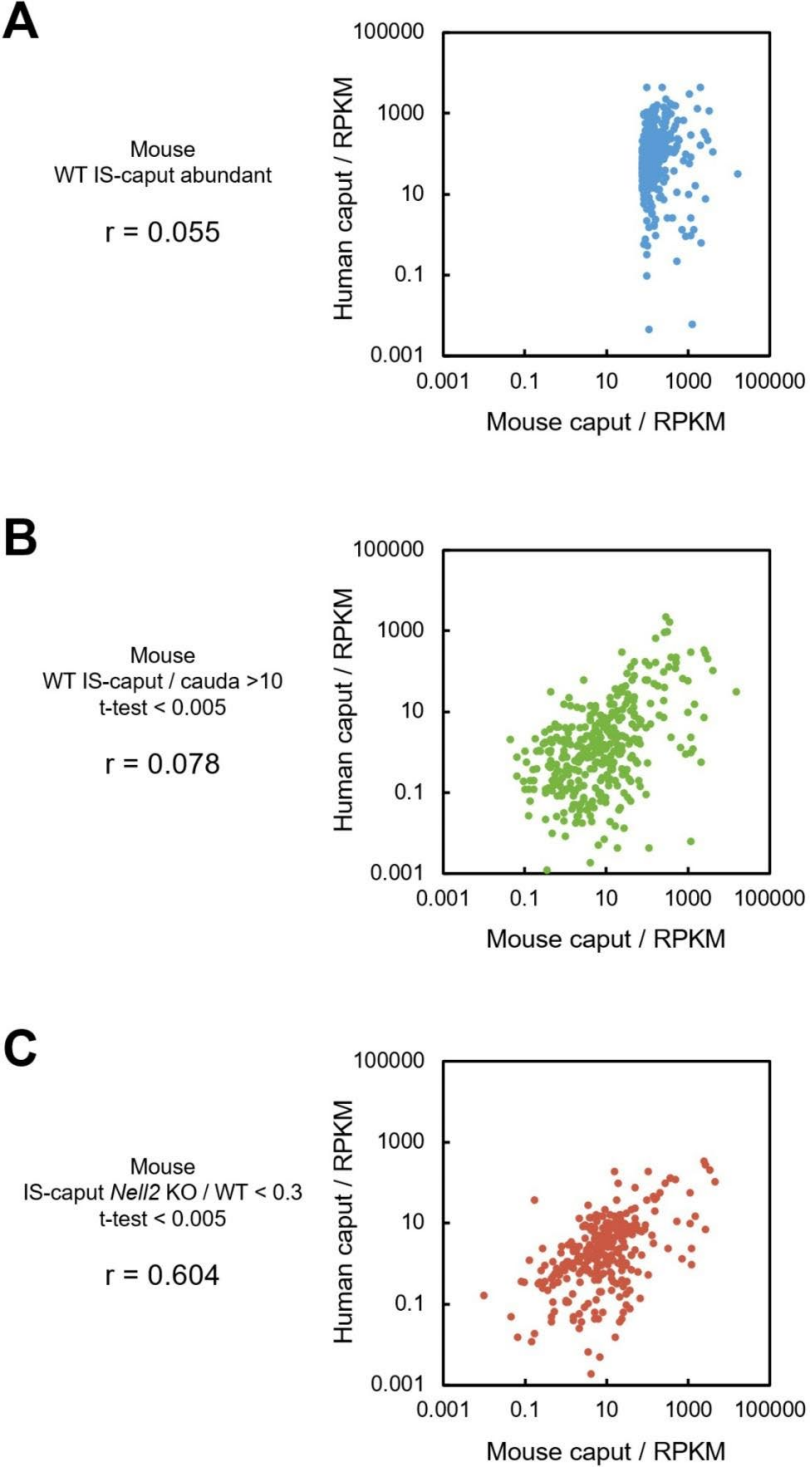
RNA-seq profiling of genes orthologous between human and mouse expressed in the caput epididymis. (**A**) Plot representation of orthologs abundantly expressed in the mouse IS-caput epididymis. (**B**) Plot representation of orthologs highly dominantly expressed in the mouse IS-caput epididymis (IS-caput/cauda > 10, t-test < 0.005). (**C**) Plot representation of orthologs whose expressions in the *Nell2* KO IS-caput epididymis are downregulated (*Nell2* KO/WT < 0.3, t-test < 0.005). The correlation coefficient r is indicated in each panel

Mouse genes expressed in the IS-caput epididymis were enriched in three different ways (I ~ III) and the human orthologs of such enriched mouse genes were identified (Table 1): (I) top 400 genes highly expressed in the WT mouse IS epididymis, 353 orthologs existed in humans, and the transcriptome data are available for 341 orthologs; (II) 450 genes relatively highly expressed in the WT mouse IS-caput epididymis (IS-caput/cauda > 100, t-test < 0.005), 372 orthologs existed in humans, and transcriptome data are available for 359 orthologs, (III) 342 genes significantly downregulated in *Nell2* KO mouse IS-caput epididymis (*Nell2* KO/WT < 0.3, t-test < 0.005), 295 orthologs existed in humans, and transcriptome data are available for 284 orthologs. The selected orthologs were represented in plots (Fig. 2a-c). The correlation coefficient r between mouse IS-caput epididymis and human caput epididymis for each group was calculated: r = 0.005, 0.078, and 0.604 for the criteria I, II, and III, respectively.

**Table 1:** A Selection of mouse genes expressed in the epididymis and their human orthologs

Criteria	Mouse genes	Human orthologs	Mouse and human data available
Mouse WT IS-caput abundantly expressed	400	353	341
Mouse WT IS-caput / WT cauda > 10 t-test < 0.005	450	372	359
Mouse *Nell2* KO IS-caput / WT IS-caput < 0.3 t-test < 0.005	342	295	284

**Fig. 3:**
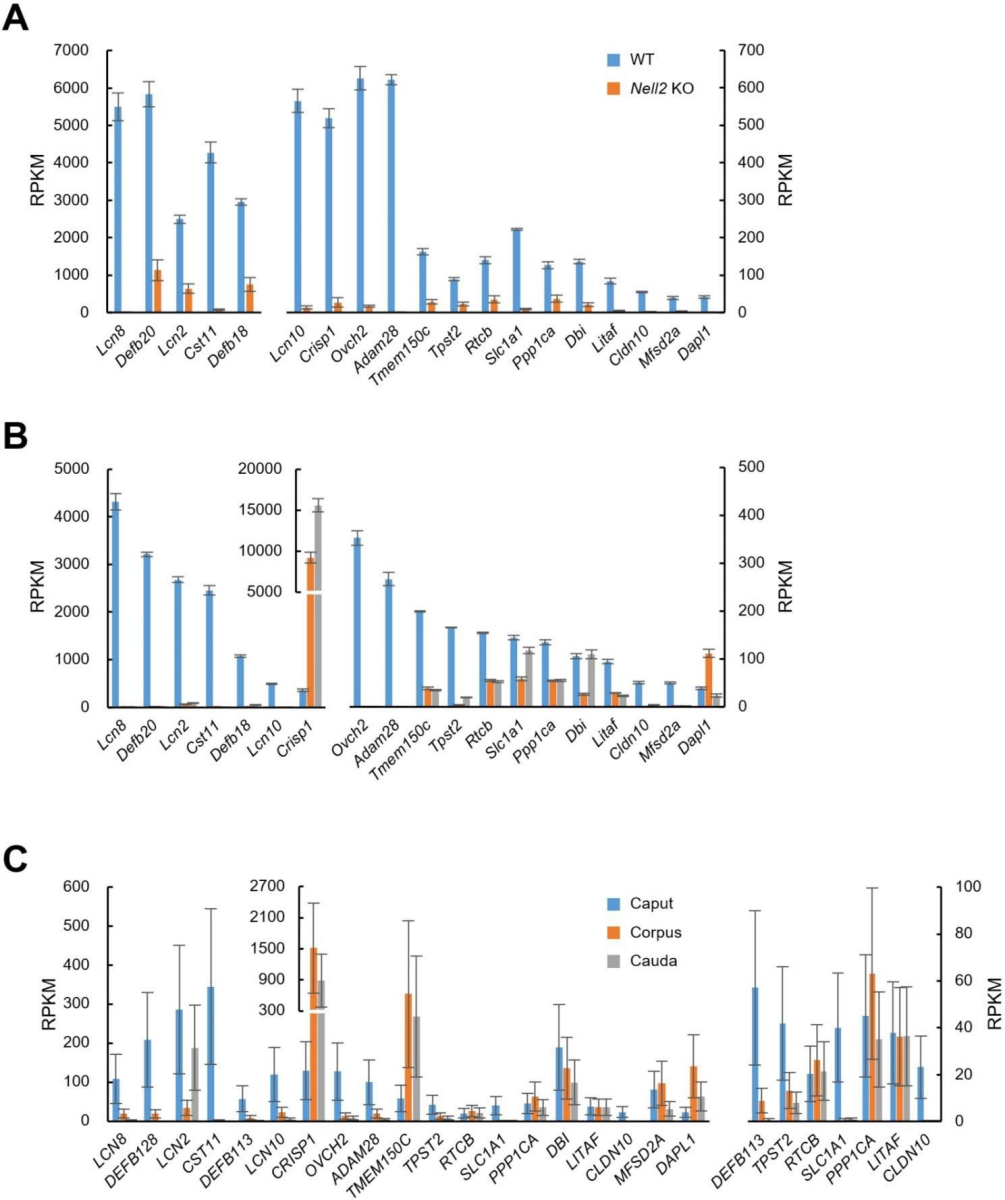
Expressions of orthologs expressed in the mouse and human caput epididymis. (**A**) Mouse gene expression in the WT and *Nell2* KO IS-caput epididymis. (**B**) Mouse gene expression in the WT IS-caput, corpus, and cauda epididymis. Note that Y axes are magnified for relatively low-expressing genes. (**C**) Human gene expression in the caput, corpus, and cauda epididymis Average ± SE is shown for 19 selected orthologs (mouse IS-caput > 20 RPKM and human caput > 20 RPKM) (left). A magnified view for relatively low-expressing genes is also shown (right)

Since the correlation constant r for selection criterion III is especially high, 19 genes, i.e., *Lcn8*/*LCN8*, *Defb20*/*DEFB128*, *Lcn2*/*LCN2*, *Cst11*/*CST11*, *Defb18*/*DEFB113*, *Lcn10*/*LCN10*, *Crisp1*/*CRISP1*, *Ovch2*/*OVCH2*, *Adam28*/*ADAM28*, *Tmem150c*/*TMEM150C, Tpst2*/*TPST2*, *Rtcb*/*RTCB*, *Slc1a1*/*SLC1A1*, *Ppp1ca*/*PPP1CA*, *Dbi*/*DBI*, *Litaf*/*LITAF*, *Cldn10*/*CLDN10*, *Mfsd2a*/*MFSD2A*, *Dapl1*/*DAPL1*, highly expressed in both human and mouse caput epididymides (mouse IS-caput > 20 RPKM and human caput > 20 reads per kilobase million [RPKM]) were picked out to interpret the result of selection based on the criterion III. The epididymal expression of these genes was visualized as follows: mouse IS-caput WT vs. *Nell2* KO (Fig. 3a), mouse WT IS-caput, corpus, and cauda (Fig. 3b), human caput, corpus, and cauda (Fig. 3c). Most mouse genes were expressed higher in the IS-caput than in corpus and cauda epididymis, and not all but many human orthologs were also expressed higher in the caput than in the corpus and cauda epididymis.

### Bioinformatic characterization of the testis-epididymis trans-luminal signaling in the human male reproductive tract

In the rodent male reproductive organs, NELL2 and NICOL are secreted in the seminiferous lumen and go from the testis through the lumen to the epididymis where they bind their receptor ROS1 to regulate IS differentiation and gene expression [9, 10]. In mice, *Nell2* and *Gm1673* encoding NELL2 and NICOL, respectively, are expressed in adluminally located cells such as the spermatocyte and spermatid subpopulations inside the seminiferous tubules and satisfy the criterion necessary for testis-epididymis trans-luminal signaling [9, 10]. To evaluate the possibility of testis-epididymis trans-luminal signaling in the human male reproductive tract, the expression of genes in the human male reproductive organs was bioinformatically explored by using single-cell RNA-seq data.

**Fig. 4:**
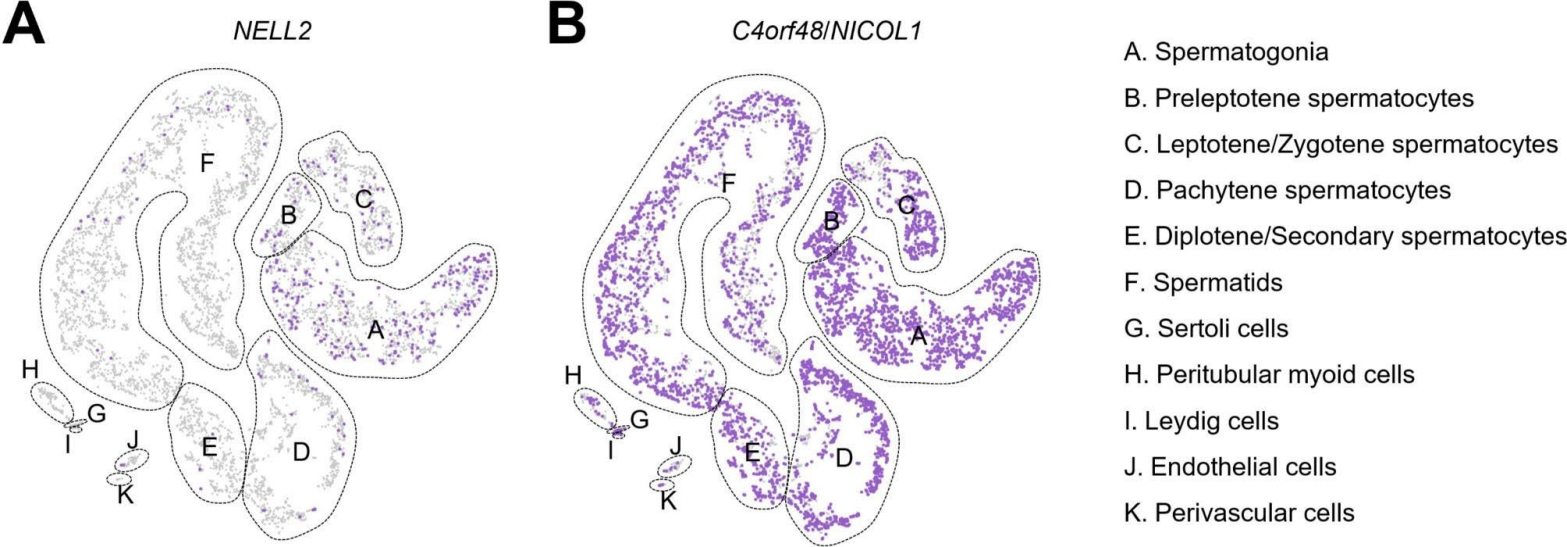
Expression profiles of *NELL2* and *NICOL* encoding human orthologs of rodent testis-epididymis trans-luminal signaling ligands. (**A**,**B**) are t-distributed stochastic neighbour embedding (t-SNE) plots showing the expression of *NELL2* (**A**) and *NICOL* (**B**) profiled by human testis single-cell RNA-seq

The human testis single-cell RNA-seq data [19] were analyzed to examine the expression patterns of *NELL2* encoding NELL2 and *C4orf48* (or *NICOL1*) encoding NICOL in the human testis. *NELL2* was expressed in the cell populations of spermatogonia, preleptotene spermatocytes, leptotene/zygotene spermatocytes, and pachytene spermatocytes (Fig. 4A). *C4orf48* was expressed in almost all cell populations (Fig. 4B). Therefore, *NELL2* and *C4orf48*/*NICOL1* were co-expressed in the cell populations of spermatogonia, preleptotene spermatocytes, leptotene/zygotene spermatocytes, and pachytene spermatocytes. Since spermatocytes at their later stages such as pachytene spermatocytes are located adluminally and their secreting proteins such as NELL2 and NICOL are included in the seminiferous fluid and able to go trans-luminally to the epididymis.

### Characterization of protein interactions mediating testis-epididymis signaling

**Fig. 5:**
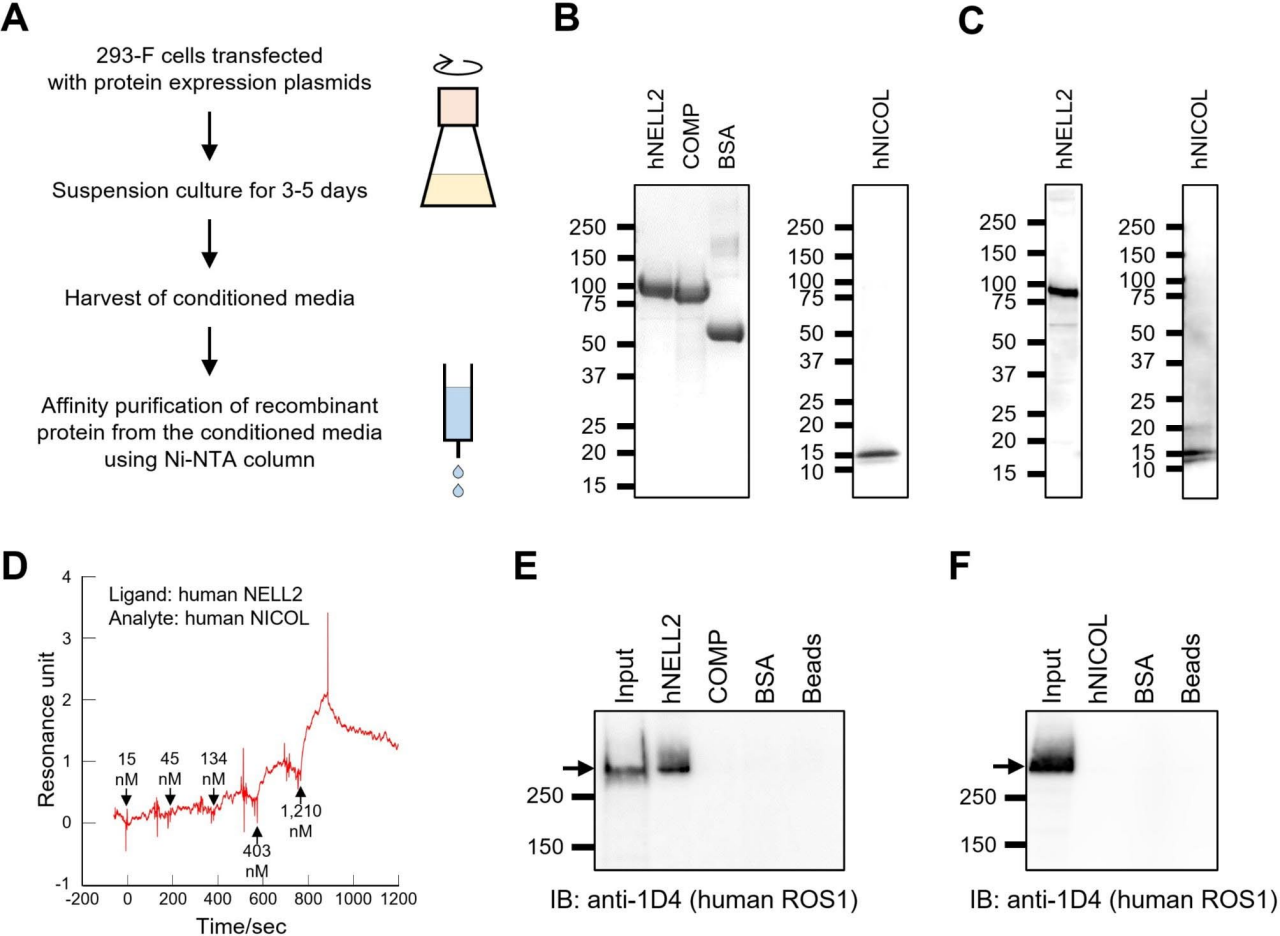
In vitro characterization of molecular functions of human NELL2 and NICOL. (**A**) A schematic representation of recombinant protein expression and affinity purification. (**B**) Coomassie brilliant blue (CBB) staining of recombinant NELL2, recombinant COMP, BSA (left) and recombinant NICOL (right) proteins. (**C**) Immunoblot detection of recombinant human NELL2 (left) and human NICOL (right) with anti-NELL2 and anti-NICOL antibodies, respectively. (**D**) The association and dissociation of recombinant human NICOL bound to recombinant human NELL2 analyzed by surface plasmon resonance analysis. (**E**) Human ROS1 pulldown with human NELL2, COMP, and BSA. (**F**) Human ROS1 pulldown with human NICOL and BSA

In the present study, molecular interaction assays using purified proteins were employed as much as possible to rigorously assess protein-protein interactions. In mice, NELL2 and NICOL constitute a ligand molecular complex [10]. The potential of interaction between human NELL2 and human NICOL was examined in vitro using recombinant proteins expressed in mammalian cells. C-terminally 6×His-tagged human NELL2 and NICOL proteins were transiently expressed in 293-F cells and purified from the conditioned media by nickel affinity chromatography (Fig. 5A-C).

The kinetic parameters of the interaction between human NELL2 and NICOL were determined by the surface plasmon resonance analysis using purified recombinant human NELL2 as a ligand and human NICOL as an analyte (Fig. 5D). The association rate constant *k*_a_ and the dissociation rate constant *k*_d_ were determined to be 9.1 × 10^3^ M^− 1^s^− 1^ and 9.8 × 10^− 4^ s^− 1^, respectively. Consequently, the dissociation equilibrium constant *K*_D_ = *k*_d_/*k*_a_ was calculated to be 110 nM, which is comparable to the *K*_D_ =80 nM for interaction between myc epitope and anti-myc monoclonal antibody 9E10 clone (*K*_D_ =80 nM) [21], indicating the tight NELL2-NICOL interaction.

Using purified recombinant proteins, the interactions of NELL2 and NICOL with ROS1 were also investigated. As the 6×His-tagged recombinant human ROS1 protein was not able to be purified probably because of the steric hindrance to the C-terminally located 6×His tag by ROS1 protein itself, we used cell lysates of 293-F cells that were transiently expressing full-length human ROS1 for the interaction assay. When the cell lysates were mixed with recombinant human NELL2, COMP, or BSA, only human NELL2 specifically pulled down ROS1 (Fig. 5E). Human NICOL did not pull down ROS1 (Fig. 5F), indicating that the NICOL-ROS1 interaction is not strong enough to be detected by the assay employed. These findings elucidate unveiled the molecular capabilities of human NELL2 and NICOL to form a complex and interact with human ROS1, particularly through NELL2.

## Discussion

In the mouse epididymis, the IS exhibits histologically prominent features characterized by pseudostratified luminal epithelium, making its regression easily observable through experimental interference of testis-epididymis trans-luminal signaling. Consequently, such histological features of the IS in mice have been employed as indicators of lumicrine control in rodents [6, 7, 9, 10, 14]. In contrast, the human caput epididymis does not exhibit pronounced luminal epithelium thickening as observed in the mouse IS epididymis [16, 17]. However, the absence of histological features akin to the mouse IS in the human caput epididymis does not provide evidence to conclude that the testis-epididymis trans-luminal signaling does not function in humans. In this study, we analyzed the possibility of testis-epididymis trans-luminal signaling in humans based on transcriptome and biochemical data.

### Genes expressed in the human epididymis and whose orthologs are regulated by testis-epididymis trans-luminal signaling in mice

In the epididymis, various proteins including protein families such as β-defensins, lipocalins, and cystatin-related epididymal spermatogenic (CRES) proteins are abundantly expressed in the proximal epididymis including the IS and the caput, to modify the fertilizing ability of spermatozoa [22]. In the present study, genes characterizing mouse IS-caput were selected in three different ways: abundant expression, IS-caput-specific expression, and lumicrine-regulated expression. The correlation coefficient r = 0.604 between IS-caput expression of the lumicrine-regulated mouse genes and caput expression of their human ortholog is significantly high compared with the others, implying a possibility that lumicrine signaling may also be active in the human male reproductive tract.

Among the orthologs thus identified and abundantly expressed in the mouse and human proximal epididymis as shown in Fig. 3, included multiple genes essential for sperm maturation and male fertility. Mice lacking 8 cystatin genes (*Cstl1*, *Cst11*, *Cstdc1*, *Cst12*, *Cst8*, *Cst13*, *Cst9*, and *Cstdc2*) on chromosome 2 possess defective sperm maturation and male infertility [9, 23]. Lipocalins (*Lcn8*/*LCN8*, *Lcn2*/*LCN2Lcn2*, *Lcn10*/*LCN10*): mice lacking 5 lipocalin genes (*Lcn5*, *Lcn6*, *Lcn8*, *Lcn9*, and *Lcn10*) clustered on chromosome 2 possess abnormal sperm maturation and male subfertility [24]. *Crisp1* KO male mice are fertile but possess reduced in vitro fertilizing ability [25]. *Ovch2* and *Adam28* encode secreted proteases. Mice lacking *Ovch2* or *Adam28* possess abnormal sperm maturation and complete infertility or reduced male fertility, respectively [9]. *Tpst2*, Altered sperm-egg interactions [26]. β-defensins constitute a large family which has evolved by gene duplication in each species and are expected to function in a compensatory and/or synergistic manner. There are no reports on whether *Defb20* and *Defb18* are essential for sperm maturation and male fertility, but deletion of nine β-defensin genes in the mouse results in defective sperm maturation and male sterility in mice [27] and the deletion of two or three β-defensin genes result in decreased sperm maturation in rats [28]. The facts support the possibility that there is a common regulatory mechanism between rodents and humans for gene expression and sperm maturation in the epididymis, although it should be confirmed experimentally further.

### Testicular cells expressing testis-epididymis trans-luminal signaling ligands

A prerequisite to the trans-luminal action of testis-epididymis signaling is the expression of ligand proteins in the testicular cells facing the seminiferous lumen. Since spermatogonial stem cells, undifferentiated and differentiated spermatogonia, and preleptotene spermatocytes are all sequestered outside the blood-testis barrier (BTB) in the basal compartment, lumicrine factor expression should take place in cells such as late spermatocytes (e.g., zygotene, pachytene, and diplotene spermatocytes), secondary spermatocytes, spermatids, or spermatozoa restricted to the apical compartment in the seminiferous epithelium [29]. The observed *NELL2* and *NICOL* expression in these cells satisfies the above criterion for testicular lumicrine factors. A preceding study demonstrating *ROS1* expression in the human epididymis by in situ hybridization and qPCR analyses [30] suggests the potential of ROS1 to function as a lumicrine receptor in humans. The dissociation constant *K*_D_ = 110 nM for human NELL2-human NICOL interaction determined by surface plasmon resonance analysis in the present study is comparable to the *K*_D_ = 87 nM for mouse NELL2-mouse NICOL interaction [9], indicating that NELL2 and NICOL form a tight heteromeric complex also in humans. The ROS1 pulldown assay using purified NELL2 and NICOL detected NELL2-ROS1 interaction, indicating that a molecular complex by NELL2 and NICOL could be a ligand for ROS1. NICOL-ROS1 interaction should be further evaluated in both mice and humans to evaluate its role in lumicrine signaling. Collectively, these results suggest that the testicular NELL2 and NICOL and the epididymal ROS1 can constitute a lumicrine trans-luminal signaling system in the human male reproductive tract. An intriguing question is whether dysfunction of NELL2, NICOL, or ROS1 causes male infertility, although mutations in these genes have not been identified as causal factors in humans.

### Evaluation of the whole processes of testis-epididymis trans-luminal signaling in the human male reproductive tract

The step-by-step processes of testis-epididymis trans-luminal signaling in the male reproductive tract of mice and humans and their supporting evidence were comparatively summarized in Table 2. Most cellular and molecular level events in testis-epididymis trans-luminal signaling were conserved between mice and humans, suggesting the possibility that trans-luminal signaling is also in humans. Whether lumicrine regulates gene expression in the proximal epididymis in humans is still unclear. However, it is worth noting that human orthologs of murine genes, which are regulated by lumicrine in the mouse epididymis, are also expressed in the human proximal epididymis.

**Table 2:** Summary of testis-epididymis trans-luminal signalling events in rodents and humans

Signalling steps	Events examined	Rodents	Humans
Testicular ligand gene expression satisfying trans-luminal signalling	*Nell2*/*NELL2* and *Nicol1*/*NICOL1* expression	Yes [9, 10]	Yes
Complex formation of testicular ligand proteins	NELL2-NICOL interaction	Yes [10]	Yes
Testicular ligand-epididymal receptor interaction	NELL2-ROS1 interaction NICOL-ROS1 interaction	Yes [9, 10]	Yes NELL2-ROS1 interaction
Epididymal gene expression regulated by testis-epididymis trans-luminal signalling	Epididymal expression of genes regulated by *Nell2*	Yes [9]	Unknown, but human orthologs of mouse lumicrine-regulated genes are expressed

It is still necessary to further examine new findings obtained from experimental animal studies with available human data on whether testis-epididymis trans-luminal signaling functions in humans and if so, to what extent it is the same and how it differs from that in experimental animals. As the volume of bioinformatic data has exploded in recent years, it is expected that the effective use of such data will lead to a further understanding of human testis-epididymis trans-luminal signaling.

### Electronic supplementary material

Below is the link to the electronic supplementary material.

Supplementary Material 1

## Data Availability

The data used and/or analysed during the current study are available from the corresponding author.

## Declarations

### Submission declaration and verification

The author declares that this manuscript has not been published previously and is not under consideration for publication elsewhere.
