# Supplementary figures and images for: Expression of NELL2/NICOL-ROS1 lumicrine signaling-related molecules in the human male reproductive tract
Source: Reprod Biol Endocrinol. 2024 Jan 2;22:3. doi: 10.1186/s12958-023-01175-6 (PMC10759339; doi:10.1186/s12958-023-01175-6)

Figure 5B

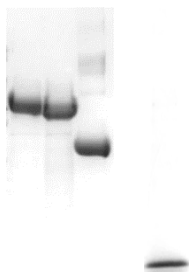

Figure 5C

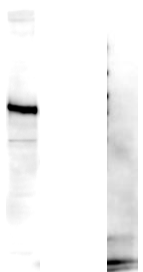

Figure 5E

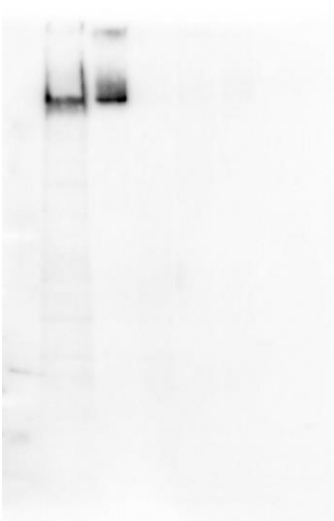

Figure 5F

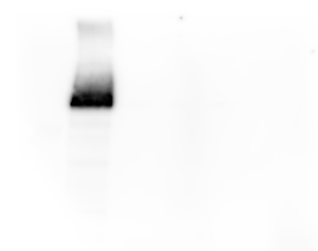

Supplement: Supplementary file 1 — Supplementary Material 1 [file 12958_2023_1175_MOESM1_ESM.pdf]
